# Development and validation of a French patient-based health-related quality of life instrument in kidney transplant: the ReTransQoL

**DOI:** 10.1186/1477-7525-6-78

**Published:** 2008-10-13

**Authors:** Stéphanie Gentile, Elisabeth Jouve, Bertrand Dussol, Valerie Moal, Yvon Berland, Roland Sambuc

**Affiliations:** 1 Department of Public Health, EA 3279, University of Aix-Marseille II, France; 2 Department of Nephrology and Kidney Transplantation, Hospital Conception, Marseille, France

## Abstract

**Background:**

In the absence of a French health-related quality of life (QOL) instrument for renal transplant recipients (RTR), we developed a self-administered questionnaire: the ReTransQol (RTQ).

**Methods:**

This questionnaire was developed using classical methodology in the following three phases over a two-year period: Item Generation phase, identifying all possible items having adverse impact on the QOL of RTR, Item Reduction phase, selecting the most pertinent items related to QOL, and Validation phase, analyzing the psychometric properties. All RTR involved in these phases were over 18 and were randomly selected from a transplant registry.

**Results:**

Item generation was conducted through 24 interviews of RTR. The first version of RTQ (85 items) was sent to 225 randomized RTR, and 40 items were eliminated at the end of the item reduction phase. The second version of RTQ (45 items) was validated from 130 RTR, resulting in the RTQ final version. The factor analysis identified a structure of five factors: Physical Health (PH), Mental Health (MH), Medical Care (MC), Fear of losing the Graft (FG) and Treatment (TR). The psychometric properties of RTQ were satisfactory. Comparison between known groups from the literature confirmed the construct validity: patients without employment or living alone have lower QOL scores, and women have lower QOL scores than men. RTQ was more responsive than SF36 to detect changes in the QOL of RTR who were hospitalized secondary to their renal disease in the 4 weeks preceding their inclusion.

**Conclusion:**

According to French public health priorities, RTQ appears to be a reliable and valid questionnaire.

## Introduction

Health-Related Quality of Life (QOL) measurements have become an important outcome measure in addition to morbidity and mortality rates, both in population health assessment and in clinical trials [1,2]. QOL indicators are based on the completion of standardized and well-validated questionnaires, addressing the impact of health status in individuals, as perceived by themselves through physical, emotional, mental, social and behavioral components [3]. Formal Quality of Life (QOL) analyses have defined the patient's role as essential to the transplant process, providing health care professionals with information regarding the psychosocial and physical impact of kidney transplantation [4,5].

Kidney transplantation is the therapy of choice for end-stage renal failure when focusing on survival transplantation [6-9] and also provides the greatest QOL, whose measurement has become an important outcome parameter [10-16].

Few specific questionnaires of QOL have been developed [17-19] for Renal Transplant Recipients (RTR), but they were not validated or available in French. Among questionnaires adapted to the general population, SF36 remains the most widely used in studies of QOL [10,20-27]. We purposefully did not make a direct transcultural validation of one of the existent questionnaires for RTR because some dimensions were lacking in these questionnaires, such as those related to medical care. Additionally, specific questionnaires, particularly the ESRS-CL [18] were, in our opinion, too centered on symptomatology and drug side effects. Lastly, existing questionnaires require face-to-face administration, when on the contrary we purposefully wished to develop a self-administered questionnaire, an important approach of this study.

This paper describes the development and validation of this questionnaire: The ReTransQol (RTQ).

## Methods

Study Design for the scale development included three phases over a two-year period:

***Phase 1: item generation***, identifying all possible items having adverse impact on the QOL of RTR,

***Phase 2: item reduction***, selecting the most pertinent items related to QOL,

***Phase 3: validation ***of the psychometric properties of RTQ.

### Patients

For each phase, RTR aged over 18 and having received their graft at least 6 months prior were included. RTR who were non-French speaking, unable to answer or lost to follow-up were excluded.

For each phase, RTR were randomly selected from the registry of the transplant center of Marseille, avoiding those included in previous phases. The study was approved by the local medical ethics committee. All patients gave informed consent to participate.

### The procedure for data collection

For each phase, the procedure of data collection varied:

***For the item generation phase***, face-to-face interviews were recorded and transcribed, collecting individual views on health perception, which identified dimensions of QOL that were most affected by renal transplantation. An interview guide was based upon a structured literature review [10]. Interviews of new patients ended when data saturation had been achieved.

***For item reduction***, questionnaires were sent to the patient's residence; non-respondents were followed-up by a second letter three weeks later, then by phone if no response. Three questionnaires were involved: RTQ V1 (first version), socio-demographic questionnaire and a clinical questionnaire, based on medical records and completed by nephrologists.

***For the validation phase***, the procedure was identical to the precedent phase, but was done twice, at the start period (M0), and 6 months later (M6); additional questionnaires were utilized (SF36 and a validated stressful life events scale).

### Data collection instruments

Except the RTQ, which is this study's specifically-developed instrument, the following instruments were used:

***SF36 ***is a generic QOL scale consisting of 36 items describing eight dimensions: Physical Function (PF), Social Function (SF), Role Function – Physical (RFP), Role Function – Emotional (RFE), Emotional Well-being (EW), Vitality (VT), Bodily Pain (BP) and General Health Perception (GHP). Each dimension ranges from 0 to 100; the higher the score, the better the perceived state of health [28].

***A validated stressful life events scale ***is a checklist of stressful life events occurring in a given time period (for the present study, period M0–M6). To complete the checklist, patients quoted the events that occurred during the period, assigning to each item a level from 0 (no stress impact) to 4 (maximal impact) [29].

***Socio-demographicquestionnaires ***included items on age, sex, living arrangement, employment status, and familial status.

***Clinical questionnaire ***included etiology of end-stage renal failure, hospital admissions in the past year, comorbidities, treatments, type of previous Renal Replacement Therapy (RRT) (hemodialysis or peritoneal dialysis), length of time on RRT, any rejection episodes, time elapsed since transplantation, and any previous unsuccessful kidney transplantation. Some questions were added to the questionnaire for the last phase of validation.

### Statistical methods

#### Item generation

Each transcript was examined independently by two researchers. Data derived from verbatim transcription and field notes were initially summarized and analyzed. Textual data were reduced to concepts through open coding and logical groups of concepts were clustered into categories, and then reorganized into a pool of items. These items were discussed by a combined group of experts and patients to test their comprehensiveness and acceptability, and later encoded.

#### Item reduction

This phase selected the most clinically relevant items, relative to response rate, inter-item correlation, and floor or ceiling effects. The items were eliminated in cases of missing values exceeding 5%, high inter-item correlation (r > 0.70), or floor or ceiling effects, homoegeneously answered on response levels (over 70% for one response level). Moreover, a first factor analysis established which of the provisional RTQ items belonged to dimensions and should be retained. Items which loaded < 0.40 for all the factors were deleted. Questions were weighted equally, and the individual's score for each of the 5 dimensions was obtained by computing each item's mean score within every dimension. A missing scale score was substituted if over half of the items in each scale were missing. All dimensions were linearly transformed to a 0–100 scale, with 100 indicating the most favorable QOL.

#### Validation

Validation of the RTQ was undertaken through the following phases:

##### Item level analysis

Feasibility was measured by using the percentage of missing values for each item and item-response distribution. Item-internal consistency was assessed by correlating each item with its dimension (using the recommended standard for correlation ≥ 0.40 [30,31]). Item-discriminant validity was assessed by determining the extent to which items correlate more highly with dimensions they are hypothesized to represent than with different dimensions.

##### Internal consistency reliability of Scale scores

Cronbach's alpha coefficients were computed to estimate the internal consistency reliability of each dimension score. A reliability of at least 0.70 is recommended to compare groups of patients [32,33].

##### Construct validity

Construct validity was examined by factor analysis with varimax rotation, which tested the underlying dimensions of the 45-item RTQ. Correlation of RTQ scales with the score of SF36's same dimensions was studied.

Known group validity explores the questionnaire's ability to show differences between patient groups with different health status and/or characteristics. We used variables identified in the literature: age, sex, employment status, familial status, BMI, treatment, comorbidities, hospitalization, and previously failed transplant [25,34,35]. We specify results quantified only for RTQ and indicate the differences found with SF36.

##### Content validity

Patients were requested to point out important domains of their life that were not mentioned in the RTQ by a final open-ended question. Their responses and comments were analyzed. Cognitive debriefing was performed with a subsample of 10 patients.

##### Reproducibility and sensitivity to change

The analyses of reproducibility and sensitivity to change were performed on patient data between the time periods M0 and M6. These patients were categorized retrospectively related to data on changes in health status and stressful life events during the period of follow-up. Physicians encoded changes in health status in three modalities: stabilization, degradation or improvement of health status. Patients were classified as undergoing a stressful life event according to their responses to the "Stressful life events scale." Two categories were formed: those with a stressful life event (coded ≥ 3), and those without (< 3).

The test-retest reliability of RTQ was assessed for patients whose health status was declared unchanged between M0 and M6, and for those without stressful events. Intraclass Correlation Coefficients (ICC) were computed between scale scores for the two assessments (≥ 0.70 considered satisfactory) [36]. Sensitivity to change was assessed for patients with a degradation or improvement of their health status and/or for those who had a stressful event between the two time periods. RTQ scores were compared using the paired t-test.

**Figure 1:**
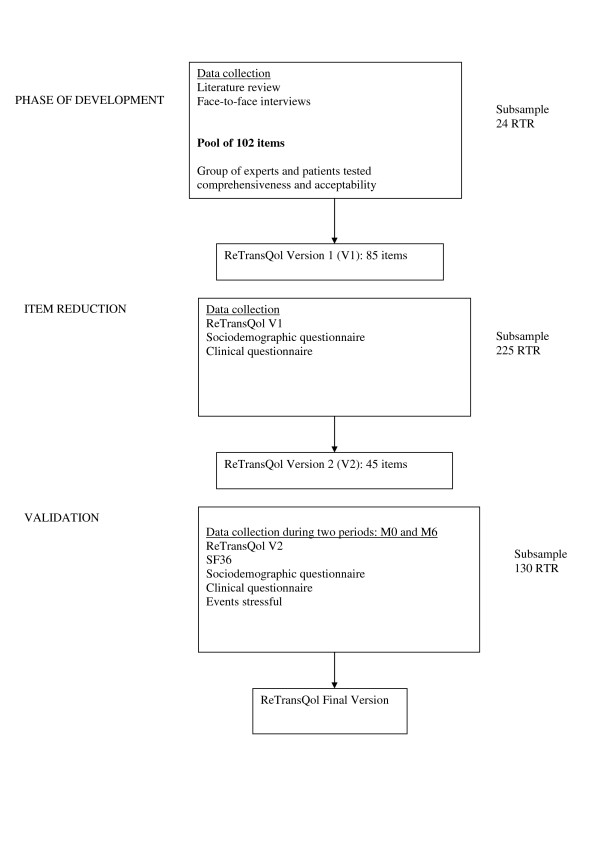
The different phases of development of ReTransQol

Figure 1 summarizes the different phases of development of RTQ.

## Results

### Item generation phase

An initial pool of 102 questions was generated by content analysis of 24 recorded interviews conducted with RTR; the QOL domains most commonly affected by renal transplant were identified. The set of 102 items was discussed by a pluridisciplinary group (nephrologists, interviewers, methodologists and patients belonging to the national association of end stage renal disease patients) to test their comprehensiveness and acceptability, prompting the rejection of 17 items. This group encoded the first version of RTQ, comprised of the provisional 85 items on a five to six-point ordinal scale, according to two reference time periods : during the previous 4 weeks, or since transplantation.

### Item reduction phase

A sample of 225 RTR was recruited for this phase, and 186 responded (response rate 82.6%). The respondents' socio-demographic and medical characteristics are presented in tables 1 and 2. Items were eliminated due to missing values (n = 23), floor or ceiling effects (n = 5), low factor loading on initial factor analysis (n = 3), or a high inter-correlation coefficient (n = 9). Finally, 40 items were rejected during this phase. Items and responses modalities are presented in Table 7.

**Table 1:** Test Sample Characteristics

	**Item reduction**	**Validation phase**
**Socio-demographic data**		
Sex, % males	62.4	63.5
Age, years, mean ± SD	50.7 ± 14.6	49.4 ± 12.8
(range)	(19 – 81)	(20 – 71)
		
**Living arrangement **:		
Live alone, %	18.6	20.2
Children, %	65.9	75.5
Patients conceived child after transplantation, %	6.5	9.7
		
**Employment status**		
Employed, %	28.5	37.5
		
**Unemployed **:		
Declared in chronic ill heath (health pension), %	37.4	36.4
Retired, %	40.5	49.1
Others, %	22.1	14.5
		
**Return to occupation after transplantation, %**	37.2	51.1

**Table 2:** Medical Data

	**Item reduction**	**Validation phase**
Period of transplantation, years	6.8 ± 3.7	5.4 ± 3.14
Previous unsuccessful kidney transplants, %	-	9.6
Patient with rejection	23.2	21.4 %
		
***Cause of ESRD***		
Chronic glomerular nephritis, %	42	40.4
Diabetic nephropathy, %	3	2.9
Vascular nephropathy, %	4.5	3.8
Interstitial nephropathy, %	13.5	10.6
Hereditary nephropathy,%	17	23.1
Unclassified nephropathy,%	20	19.2
		
***Different modalities of dialysis***		
Hemodialysis	97.3	96.2
Peritoneal dialysis	2.7	3.8
Duration of dialysis, years, mean ± SD	2.5 ± 2	3.8 ± 4.2
		
***Comorbidities***		
Hypertension	78.8	88.5
Diabetes	13	13.5
Hepatitis	0.6	6.7
Peripheral vascular disease	0.6	5.8
Depression	-	3.8
Smoking status	36.5	31.1
BMI, kg/m^2^, mean ± SD	25.5 ± 4.7	24.5 ± 4.0
Comorbidity index, mean ± SD	1.6 ± 0.9	1.5 ± 0.8
		
***Treatments***		
Calcineurin inhibitors	94.6	96.2
Corticosteroids	89.3	97.1
Antimetabolites	75.3	77.9
Antihypertensive drugs	87.1	90.4
Antilipemic agents	53.2	45.2
Antidiabetic agents	9.7	15.4
Antithrombotics	28.5	29.8
Protease Inhibitors	19.9	26.0
Erythropoietin therapy	12.2	10.6
		
**Hospital admissions**		
During the last 12 months	-	45.2
Related to Kidney Transplantation	-	66.0

### Validation phase

A sample of 130 RTR, different from those involved in the item reduction phase, was randomly chosen for the validation step study; 104 patients were included (response rate 80%, Tables 1 and 2).

#### Item level analysis and internal consistency reliability scores

In accordance with the results of item selection, all response levels of each item were homoegeneously answered. At the item level, missing data did not exceed 5%. The acceptability of RTQ was satisfactory (77% completion). Table 3 presents results of internal item consistencies.

**Table 3:** Internal Consistencies

**ReTransQOL** **Dimensions**	**Mean ± SD** **(Min – Max)**	**Cronbach's α** **coefficient** **(range if item was deleted)**	**IICa** **(Min – Max)**	**IIC** **% > 0.40**	**IDVb**	**IDV** **% < 0.40**
Physical Health (PH)	58.5 ± 19.2 (13.9 – 95.3)	0.86 (0.84 – 0.86)	0.48 – 0.73	100%	0.07 – 0.50	92.5%
Mental Health (MH)	69.3 ± 17.3 (4.9 – 100)	0.84 (0.80 – 0.85)	0.38 – 0.71	88.9%	0.04 – 0.48	83.3%
Medical Care (MC)	64.4 ± 11.0 (37.3 – 84.8)	0.83 (0.79 – 0.83)	0.44–0.70	100%	0.01 – 0.44	97.7%
Fear of losing the graft (FG)	53.8 ± 20.9 (3.6 – 96.4)	0.79 (0.75 – 0.78)	0.64 – 0.78	100%	0.07 – 0.53	95.8%
Treatment (TR)	65.8 ± 15.5 (23.1 – 94.8)	0.70 (0.66 – 0.71)	0.37 – 0.66	77%	0.01 – 0.49	91.6%

a ICC, Internal Item Consistency correlation coefficient: Range of correlations between items and their dimension

b IDV, Item Discriminant Validity : Range of correlations between items and other dimension

**Table 7:** Items and responses modalities

**Item number**	**Items label**	**Response modalities**
1	Have you had physical pain?	All most time
2	Has your graft bothered you?	Most of time
3	Have you felt tired?	A good bit of the time
4	Do you engage in physical exercise?	Some of the time
5	Do you feel energetic?	A little of the time
		None of time

6	You are as well as anyone else	Definitely agree
7	You have stopped doing certain things.	Mostly agree
8	You feel autonomous.	Not agree not disagree
9	You can do your housework and errands by yourself.	Mostly disagree
		Definitely disagree

10	Do you feel physically affected?	
11	Are you annoyed by the side effects of treatment?	
12	Is your weight a problem for you?	
13	Do you feel relieved?	
14	Do you feel sick?	No at all
15	Have you been able to forget that you received a graft?	A little bit
16	Do you often think about your graft?	Moderately
17	Are you satisfied with your graft?	Quite a bit
18	Does your family offer you moral support?	Extremely
19	Has your family accepted your illness?	
20	Do you feel misunderstood by the people around you?	
21	Do you feel close to your friends?	

22	Do you feel sad?	
23	Have you enjoyed life as much as possible?	
24	Do you feel discouraged?	All most time
25	Are you able to put up with daily worries and stress?	Most of time
26	Do you feel isolated?	A good bit of the time
27	Are you anxious about your state of health?	Some of the time
28	Does waiting for the results of medical tests distress you or make you feel scared?	A little of the time
29	Do you think about a possible return to dialysis?	None of time
30	Do you still sometimes think about dialysis?	

31	You would say that you have a normal life.	Definitely agree
32	You live with the graft as if you have a second life, a rebirth.	Mostly agree
33	Do you think you will have enough income to provide for your needs?	Not agree not disagree
		Mostly disagree
		Definitely disagree

34	Do you have hobbies/leisure activities?	All most time
		Most of time
		A good bit of the time
		Some of the time
		A little of the time
		None of time

35	Is taking medications a constraint for you?	
36	Are you scared of the possible side effects of the anti-rejection treatment?	
37	Are your doctor's orders restrictive?	
38	Do you trust your nephrologist?	
39	Do you have trust in the prescribed treatments?	
40	Are you satisfied by your nephrologist's ability to listen?	No at all
41	Do you feel sufficiently informed by your nephrologist?	A little bit
42	Do you feel like you are sufficiently informed about the side effects of your treatments?	Moderately
43	Do you feel like you are sufficiently informed about complications of the graft?	Quite a bit
44	Do you feel supported by the medical team?	Extremely
45	Are you satisfied by your medical follow-up?	

#### Construct validity

##### - Factor analysis

The factor analysis with varimax rotation identified a structure of five factors, which accounts for 46.3% of the total variance (Table 4). The content of each dimension was entitled the following: Physical Health (PH, ten items), Medical Care (MC, eleven items), Fear of losing the Graft (FG, six items), Treatment (TR, nine items) and Mental Health (MH, nine items).

**Table 4:** Dimension and factor loadings identified using principal component analysis and the orthogonal varimax rotation (only factor loading >0.3 are mentioned)

	**Factors**					
***Dimensions Labels***	**1**	**2**	**3**	**4**	**5**	**Item number**
Eigenvalues	9.25	4.10	2.99	2.43	2.08	
% of variance	20.56	9.12	6.65	5.4	4.6	

***Physical Health- PH***	**.726**					q5
	**.674**					q6
	**.641**					q36
	**.588**			.355		q7
	**.573**					q8
	**.551**					q4
	**.538**			.313		q14
	**.536**					q9
	**.508**					q33
	**.305**					q35

***Medical Care – MC***		**.767**				q43
		**.737**				q42
		**.708**				q40
		**.645**				q41
		**.633**				q49
		**.608**				q45
		**.574**				q44
		**.544**				q46
	.327	**.370**				q34
	.474	**.330**				q13
	.387	**.307**				q17

***Fear of losing graft – FG***			**.719**			q31
			**.709**			q32
			**.689**			q16
			**.668**			q30
			**.622**			q15
			**.561**	.338		q29

***Treatment – TR***			.327	**.305**		q39
	.389			**.617**		q1
				**.569**		q11
				**.565**		q12
	.536			**.547**		q3
			.356	**.546**		q38
				**.524**		q10
				**.394**		q2
		.304		**.379**		q37

***Mental Health – MH***					**.671**	q28
					**.657**	q19
					**.650**	q21
					**.647**	q20
					**.574**	q22
	.464			.308	**.512**	q23
	.500				**.404**	q26
	.478				**.312**	q27
	.595				**.432**	q24

##### - Correlation between SF36 and RTQ

Positive correlations were found between RTQ scores and SF36 scores. The dimension scores of RTQ had medium to high correlation (> 0.6) with those of SF36 assessing similar dimensions: PH-RTQ with PF-SF36, RFP-SF36, VT-SF36, and MH-RTQ with EW-SF36. The other RTQ dimension scores: MC-RTQ, FG-RTQ and TR-RTQ were not highly correlated with SF36 (Table 5).

**Table 5:** Correlation between RetransQol and SF36

	**ReTransQol**				
**SF36**	**Physical Health**	**Mental Health**	**Medical Care**	**Fear of losing the Graft**	**Treatment**

Physical Function- PF	0.680**	0.445**	0.268**	0.133	0.387**
Social Function- SF	0.641**	0.605**	0.184	0.290**	0.489**
Role Function-Physical-RFP	0.554**	0.450**	0.153	0.085	0.276**
Role Function-Emotional-RFE	0.612**	0.618**	0.241**	0.190	0.389**
Emotional Well-being – EW	0.543**	0.638**	0.235**	0.385**	0.474**
Vitality- VT	0.746**	0.559**	0.230*	0.366**	0.593**
Bodily Pain- BP	0.564**	0.451**	0.113	0.163	0.546**
General Health Perception-GHP	0.760**	0.498**	0.318**	0.356**	0.497**
Physical Score Composite	0.635 **	0.374**	0.117	0.101	0.388**
Psychological Score Composite	0.641**	0.760**	0.223	0.375**	0.546**

** p < 0.001

##### - Known Group Validity

Table 6 presents a summary of variables associated with a decreased or increased QOL.

**Table 6:** Known Group Validity

	**ReTransQol**					**SF36**							
	
	**PH**	**MH**	**MC**	**FG**	**TR**	**PH**	**SF**	**RF**	**RE**	**MH**	**V**	**BP**	**GH**
**Variables associated with a decreased quality of life**													

Unemployed	+	+				+	+	+	+		+	+	+

Living alone		+											

Female					+								

Age > 55						+	+	+	+			+	

Hospitalizations in previous 12 months	+					+		+	+				+

Hospitalizations in previous 12 months for KT	+		+										

Period of transplantation					+							+	

Comorbidities index		+		+		+							+

Diabetes			+	+									

Depression		+			+		+						

BMI	+												

Smoking status	+												

Stressful life events	+	+					+			+	+		

**Variables associated with increased quality of life**													

Previous unsuccessful Kidney transplants				+	+						+		+

Antimetabolites					+								

+ = Indicates a statistically significant relationship

Patients living alone reported significantly lower scores on the dimension MH (63.2 ± 2.2 vs. 71.4 ± 14.9, p < 0.05). Patients without employment reported lower scores on dimension PH (53.3 ± 19 vs. 65.6 ± 16.9, p < 0.05) and MH (65.8 ± 17.7 vs. 74.3 ± 15.4, p < 0.05), and for all dimensions of SF36 except EW. Women reported lower scores on dimension TR (62.90 ± 14.7 vs. 67.9 ± 15.6, p < 0.05). Measures of RTQ were not influenced by age, yet patients over 55 reported significantly lower scores for SF36 on dimensions PF, SF, RFP, RFE and BP.

The RTR hospitalized during the previous 12 months reported significantly lower scores on dimension PH for RTQ (51.9 ± 18.8 vs. 63.9 ± 17.9, p < 0.01) and for SF36 on dimension PF, RFP, RFE and GHP. However, patients hospitalized for transplant complications reported significantly lower scores, for RTQ only on the dimensions PH (51.8 ± 18.8 vs. 69.96 ± 10.2, p < 0.01) and MC (60.7 ± 12.2 vs. 65.9 ± 10.2, p < 0.01), and not for SF36. The period of time since transplantation was significantly correlated with the score of RTQ on the TR dimension (r = 0.22, p < 0.01) and score of SF36 on the BP dimension.

RTR with a previous unsuccessful kidney transplant reported higher scores for RTQ on dimension FG (66.7 ± 16.7 vs. 52.4 ± 20.9, p < 0.05) and dimension TR (79 ± 6.6 vs. 64.3 ± 15.5, p < 0.01), and for SF36 on dimensions VT and GHP. Measures of RTQ and SF36 were neither influenced by the kind of dialysis, nor the duration of dialysis, nor a possible rejection episode.

Considering RTQ-specific results, Body Mass Index showed a significant negative correlation with RTQ score on the dimension PH (r = -0.258, p < 0.05), and smokers reported lower scores on the dimension PH (51.5 ± 19.7 vs. 63.1 ± 19.5, p < 0.01). Patients with Diabetes Mellitus reported significantly lower RTQ scores on dimension MC (38.4 ± 21.2, p < 0.05) and FG (38.4 ± 21.2 vs. 58.9 ± 19.7, p < 0.01). No difference was found for the SF36 in all of these characteristics.

Patients with a stressful life event reported lower scores on RTQ for the dimension PH (56.4 ± 20.3 vs. 68.8 ± 17.2, p < 0.01) and MH (65 ± 10 vs. 74.2 ± 16.7, p < 0.021), and lower scores for SF36 for the dimensions SF, EW, and VT.

#### Content validity

A cognitive debriefing was performed with a group of 15 RTR, members of the national association of End-Stage Renal Disease patients. The group confirmed the pertinence of the five dimensions, and the relevance of the items. The dimension of "Medical Care," which is not evoked in other QOL RTR-specific questionnaires, seems to be of high importance in relation to the patients' QOL.

#### Reproducibility and sensitivity to change

For patients estimated as clinically stable between M0 and M6 (n = 56; 83.6%), high correlation coefficients (CC) between scale scores for the two assessments were all significant (p < 0.001): PH = 0.82, MH = 0.73, MC = 0.63, TR = 0.76 and FG = 0.76.

For patients without stressful life events between M0 and M6 (n = 29; 43.3%), high CC between scale scores for the two assessments were significant (p < 0.001) for four dimensions: PH = 0.80, MH = 0.70, TR = 0.79 and FG = 0.69. For the dimension MC, CC is lower (0.380) but significant (p < 0.019).

For patients who were clinically stable and without stressful life events (n = 23; 34.3%), the CC are also high and significant between M0 and M6 (p < 0.001) for four dimensions (PH = 0.79, MH = 0.72, TR = 0.82 and FG = 0.68), and lower for the dimension MC (0.39), but significant (p < 0.027).

Among the 67 patients followed between M0 and M6, 8 patients showed deterioration in health status, 38 patients experienced a stressful life event and 4 patients showed both of these characteristics. Significant differences were neither found for the five dimensions of RTQ nor dimensions of SF36 among any of these groups of patients.

## Discussion

The psychometric properties of RTQ are satisfactory with an exception for the sensitivity to change, due to the low number of subjects with change in health status during the period of the study. Subject acceptability was excellent with a low percentage of missing data. The five dimensions were confirmed by the results of the principal component analysis. Some items (nine out of forty-five) had, for their specific dimension, a factor loading under the recommended threshold of 0.40 [30,31] and/or cross-loading. Nevertheless, they were retained due to their clinical relevance in terms of content validity. For the same reasons, the item "stress" (Q23) remained in the MH dimension, despite its higher loading in the PH dimension. This classification provided better results for reliability, content validity and clinical validity.

The RTQ revealed specific dimensions of QOL in renal transplant recipients (RTR). The dimensions "Physical Health" (PH) and "Mental Health" (MH) of the RTQ are similar to those of the SF36, but three other dimensions give specificity to the questionnaire: Fear of losing the Graft (FG), Treatment (TR) and Medical Care (MC). These concerns are found in other questionnaires published for RTR [17-19], but generally not individualized as specific dimensions. For example, the fear of losing the graft is included in the Mental Health dimension in Franke's questionnaire, the ESRD Checklist [18], and is a specified dimension in Laupacis' questionnaire entitled "Uncertain/Fear" [19]. In the same way, items concerning treatment are always present, though often limited to the side effects of drugs. For example, Laupacis' questionnaire presented a dimension called "Appearance," which specified the adverse effects of immunosuppressive medication like excessive hair growth, excessive appetite, weight and acne [19]. In Franke's questionnaire, treatment is present in two dimensions called "side effects of corticosteroids" and "increased growth of gums and hair," which are two specific effects of calcineurin inhibitors [18]. Conversely to these questionnaires, RTQ proposes a dimension of treatment which is more holistic. We think that questionnaires which list side effects, many of which are not specified, could possibly become obsolete as treatments evolve. Instead, we included items about the embarrassment caused by the side effects of drugs, and questions about the difficulties of compliance. Finally, the patient's relationship with both the doctor and the medical team ("Medical Care" of RTQ) is not dealt with by other validated questionnaires, even though patients attribute importance to medical information and health education.

Comparisons between different demographic subgroups confirm previous empirical works showing their variations [25,34,35]. For example, results confirm that patients without employment or living alone have lower QOL scores. Also, women have lower QOL scores in comparison with men, only for the dimension of "treatment." This is probably in relation to the impact of immunosuppressive treatments on the body image [18,25,34,35].

RTQ was more responsive to detect changes in QOL for patients hospitalized for renal disease, for those with a high BMI, or comorbidities, especially Diabetes Mellitus. Although age was found in the literature to be a predictive factor of QOL, no significant correlation was found between RTQ and age. But most QOL studies focusing on RTR have assessed QOL with generic questionnaires [10], and among studies with QOL-specific questionnaires, its correlation with age is not precisely discussed [17-19]. Probably age is far more a general determinant of quality of life than it is specific to kidney transplantation. The RTQ can be utilized alone or in complement with the SF36, according to the clinician's objective. The interest of using the SF36 in complement with RTQ essentially is to be able to compare levels of QOL between RTR and other groups of patients (from different countries, or with other pathologies, etc.).

## Conclusion

This study reported the stages of development and validation of a QOL questionnaire for RTR, developed in response to a lack of any validated instrument for those patients in the French language. The quality of life for patients with chronic diseases has become a public health priority in France, especially since the new Public Health Law [37].

Psychometric properties allow this questionnaire to be used for monitoring the impact of different treatments on the renal transplant population, more effectively than previously possible. In addition, regular use of the RTQ can complement current evaluation of "good transplantation practice," while highlighting problem areas for specific intervention. We envision continuing this work by the follow-up of a prospective cohort to study the sensitivity to change and the clinically significant threshold. In the future, we plan to validate the questionnaire in English.

## List of abbreviations

BMI: Body Mass Index; CC: Correlation Coefficient; ESRD: End of Stage Renal Disease; QOL: Health-related Quality of life; ICC: Intraclass Correlation Coefficients; RRT: Renal Replacement Therapy; RTR: Renal Transplant Recipients; RTQ: ReTransQol; SF-36: "Short Form – 36" questionnaire. 

The abbreviations for the dimensions of RTQ are PH: Physical Health; MH: Mental Health; MC: Medical Care; FG: Fear of losing the Graft; TR: Treatment. 

The abbreviations for dimensions of SF36 are PF: Physical Function; SF: Social Function; RFP: Role Function-Physical; RFE: Role Function-Emotional; EW: Emotional Well-being; VT: Vitality; BP: Bodily Pain; GHP: General Health Perception.

## Competing interests

The authors declare that they have no competing interests.

## Authors' contributions

SG conceived the study and its design, coordinated the data management, analyzed and interpreted the data, drafted the manuscript; EJ participated in the design of the study, collected the data and performed the statistical analysis BD and VM: participated in the design of the study, collected medical data and participated to the interpretation of data RS et YB revised the manuscript critically for important intellectual content and have given final approval of the version to be published

All authors read and approved the final manuscript.
